# We need to act now

**DOI:** 10.7554/eLife.59636

**Published:** 2020-06-05

**Authors:** Michael B Eisen

**Affiliations:** Department of Molecular and Cell Biology and HHMI, University of California, BerkeleyBerkeleyUnited States

**Keywords:** racism in science, equality, diversity and inclusion, scientific publishing, eLife, careers in science, discrimination

## Abstract

eLife, like the rest of science, must tackle the many inequalities experienced by Black scientists.

I do not know how any white American like myself can respond to the murder of George Floyd by police officers in Minneapolis, Minnesota with anything but sadness, horror, and above all else, an abject sense of failure. We have, obviously, failed as a society when our social structures not only fail to prevent – but in many ways actively encourage – atrocities like this. And we have failed as individuals and organizations by not transforming the anger and sadness we felt after the deaths of Breonna Taylor, Botham Jean, Stephon Clark, Philando Castile, Freddie Gray, Tamir Rice, Michael Brown, Eric Garner and many others into any kind of meaningful action.

I know it matters to some people to hear people like me say the right things in response: that Black Lives Matter; that racism is a malignancy; that we want science to be a safe and welcoming place for Black scientists; and that we are all complicit when the power of the state is used to enforce a racist social structure by killing Black Americans. I think and feel all of these things. But I also feel like a charlatan in saying them. Because it is not the first, or the second, or the third time I have done so, and what do these words mean if they do not lead those saying them to do anything tangible in response?

The disconnect between words and actions is everywhere, but as scientists we should be focusing a particularly harsh eye on our institutions and on our individual roles in supporting the systemic racism that pervades them. And I will start with myself. I like to think of myself as a progressive who has made a persistent effort to promote diversity in science. But of the 50 graduate students and postdocs I have trained, none are Black. I have volunteered in diversity efforts at my university and elsewhere, I have sat on diversity panels, and I have reviewed diversity fellowships. But none of the many faculty search committees that I have served on made an offer to a Black candidate.

One of the things that drew me to pursue the job as Editor-in-Chief at eLife was the interest the organization had shown in promoting diversity in science. When I took over last year, I immediately began to address the lack of diversity on our editorial boards. You can judge the success of our efforts how you will, but we have unquestionably failed when it comes to race. The entire leadership team of eLife is white. We have no Senior Editors who are Black. And despite the rapid growth of our Board of Reviewing Editors, very few are Black. (See this blogpost for more details). So inured are we to the exclusion of Black colleagues from the halls of science that many of us do not notice it at all, despite claiming to want racial justice.

It is easy to make excuses – the legacy of historic racism is so strong that there are not a lot of senior Black scientists to choose from, and those that have survived the gauntlet are in demand and overcommitted, and so on and so forth. But these excuses are lame. How can we think that scientists can figure out a way to cure COVID, but not figure out a way to increase the number of Black scientists and involve them as leaders in the field? The reality is this IS a solvable problem: we have just chosen not to solve it. 

It is impossible for eLife to be true to our mission of encouraging and recognizing the most responsible behaviors in science without also becoming a model for dismantling white supremacy in science.

We will begin by recruiting Black scientists at all levels of the organization and empowering them in planning and decision making. But that is obviously not enough. There is ample evidence that the entire system of science evaluation of which eLife is a part is structurally biased against Black scientists, and that significant changes are required to fix it. Where we can take tangible action now, we will: for example, we will ensure that our scope, policies and editorial board composition lead to fair consideration of work from fields with higher concentrations of Black scientists (Hoppe et al., 2019). And we will build on ongoing efforts to root out implicit bias in our peer review and editorial decision-making processes, working with experts on bias in science evaluation to identify additional areas where we are directly or indirectly biased against Black scientists, and determine how best to respond.

However there is the real possibility that a system built around dispensing limited markers of prestige is fundamentally incompatible with true fairness, and cannot be fixed by restructuring. This is one of the reasons I have long advocated scrapping the journal system and rebuilding science publishing from the ground up. But I am conscious that a lack of bias is not the default state in new systems, and that we must be eternally vigilant in making the elimination of bias our top priority.

We will begin by recruiting Black scientists at all levels of the organization.

It might seem weird to be talking about science publishing with people being murdered on the streets. But the deep and pervasive problems in policing in the United States do not exist in isolation – they are product of the ongoing legacy of white supremacy that pervades all aspects of society – and science is no exception. We need direct and decisive action to end police violence targeted at Black Americans. But we also need to eliminate the societal structures that fuel and support it. 

I know that people will be skeptical about our willingness to deliver on these promises – and empirical evidence suggests they should be. None of these words matter if we fail to turn them into action. White scientists of the world, it really is time for us to put up or shut up. 
